# The mediating role of vitamin D in the relationship between triglyceride glucose index and mortality in patients with diabetes mellitus: a causal mediation analysis

**DOI:** 10.3389/fnut.2024.1492647

**Published:** 2024-12-18

**Authors:** Fan Zhang, Wenjian Li

**Affiliations:** ^1^Changzhou Clinical College, Xuzhou Medical University, Changzhou, China; ^2^Department of Endocrinology, Changzhou Third People's Hospital, Changzhou, China; ^3^Department of Clinical Nutrition, Changzhou Third People's Hospital, Changzhou, China; ^4^Department of Urology, Changzhou Third People's Hospital, Changzhou, China

**Keywords:** diabetes mellitus, triglyceride glucose index, vitamin D, mortality, mediation analysis

## Abstract

**Objective:**

This study investigated the effects of triglyceride glucose index (TyG) and vitamin D levels on all-cause and cardiovascular mortality in diabetic patients and assessed the potential mediating role of vitamin D in the relationship between TyG and mortality.

**Methods:**

The study was based on data from the National Health and Nutrition Examination Survey (NHANES) database from 2001 to 2018, which included 6,318 patients with diabetes. Multivariable Cox proportional risk regression models were employed to assess the association between TyG and vitamin D levels and the risk of death in diabetic patients. The interaction between TyG and vitamin D and its effect on mortality was explored through restricted cubic spline analysis and causal mediation analysis.

**Results:**

The results demonstrated that the TyG index was positively associated with all-cause and cardiovascular mortality in diabetic patients, whereas vitamin D levels were negatively associated with mortality, exhibiting an overall U-shaped association. The results indicated that vitamin D partially mediated the association between TyG and all-cause mortality. Further analysis revealed a significant mediation between vitamin D and TyG, whereby alterations in vitamin D levels influenced the impact of TyG on mortality. Subgroup analyses demonstrated that the correlation between TyG and mortality was more pronounced in diabetic patients with vitamin D insufficiency.

**Conclusion:**

The study demonstrates the mediating influence of vitamin D on the relationship between TyG and mortality in diabetic patients. This finding underscores the necessity of evaluating the influence of vitamin D on survival outcomes in individuals with disparate levels of the TyG index.

## 1 Introduction

Diabetes mellitus (DM) is a global metabolic disease characterized by elevated blood glucose levels. These levels are primarily the result of insufficient insulin secretion or a weakened cellular response to insulin (1). DM not only affects an individual’s glucose metabolism but is often accompanied by cardiovascular disease risk factors such as dyslipidemia and hypertension. These factors significantly increase the risk of cardiovascular disease and death in patients with diabetes (2). In recent years, the triglyceride glucose index (TyG), a novel clinical surrogate for insulin resistance, has garnered considerable attention in assessing diabetes risk and prognosis. This is mainly due to its simplicity, accessibility, and reproducibility (3). The TyG index effectively reflects the state of insulin resistance by combining fasting plasma glucose (FPG) and triglyceride (TG) levels. Moreover, it has been confirmed to be closely associated with the risk of developing DM and its cardiovascular complications in numerous studies (4–6).

Vitamin D, a fat-soluble vitamin, plays a role in calcium and phosphorus metabolism within the human body, thereby influencing bone health. Additionally, it affects glucose-lipid metabolism and immune function through various mechanisms (7). The primary form of vitamin D in the human body is 25-hydroxyvitamin D (25 (OH)D). The serum 25 (OH) D level can be utilized as an indicator for evaluating the deficiency, insufficiency, or sufficiency of vitamin D (8). In recent years, an increasing number of studies have demonstrated significant associations between vitamin D levels and glycemic control, dyslipidemia, cardiovascular disease risk, and mortality in diabetic patients (9, 10). In particular, vitamin D deficiency or insufficiency may increase insulin resistance, elevating the risk of diabetes and its associated complications (11, 12). Moreover, vitamin D may influence triglyceride levels by modulating the expression of genes involved in lipid metabolism, thereby indirectly impacting the TyG index (13). Some studies have demonstrated a negative correlation between vitamin D levels and the TyG index, indicating that vitamin D may enhance the prognosis of diabetic patients by influencing insulin resistance and glucolipid metabolism (14). Vitamin D supplementation may improve glycemic control and reduce the risk of diabetes-related complications in diabetic patients (11). Consequently, vitamin D levels may be a mediator in influencing the relationship between the TyG index and mortality in diabetic patients.

While studies have been conducted to investigate the relationship between vitamin D, the TyG index, and the prognosis of diabetic patients separately (15, 16), studies on whether vitamin D levels mediate the relationship between the TyG index and mortality in diabetic patients are scarce. In particular, the predictive value of the TyG index for the risk of mortality in diabetic patients under different vitamin D status and the underlying mechanisms remain to be further elucidated. This knowledge gap limits our comprehensive understanding of the prognostic assessment of diabetic patients and hampers the development of intervention strategies for at-risk populations.

In light of the existing literature and theoretical background, the present study proposes the following hypothesis: vitamin D levels may mediate the relationship between the TyG index and mortality in diabetic patients, with this effect varying across vitamin D levels. To test this hypothesis, this study will investigate the mediating role of vitamin D levels in the relationship between TyG index and mortality in diabetic patients by analyzing data from the National Health and Nutrition Examination Survey (NHANES) database. Furthermore, the predictive value of the TyG index for mortality risk under different vitamin D statuses will be analyzed. This study aims to provide a new perspective on the prognostic assessment of diabetic patients and provide a scientific basis for developing targeted interventions.

## 2 Materials and methods

### 2.1 Study population

**Figure 1:**
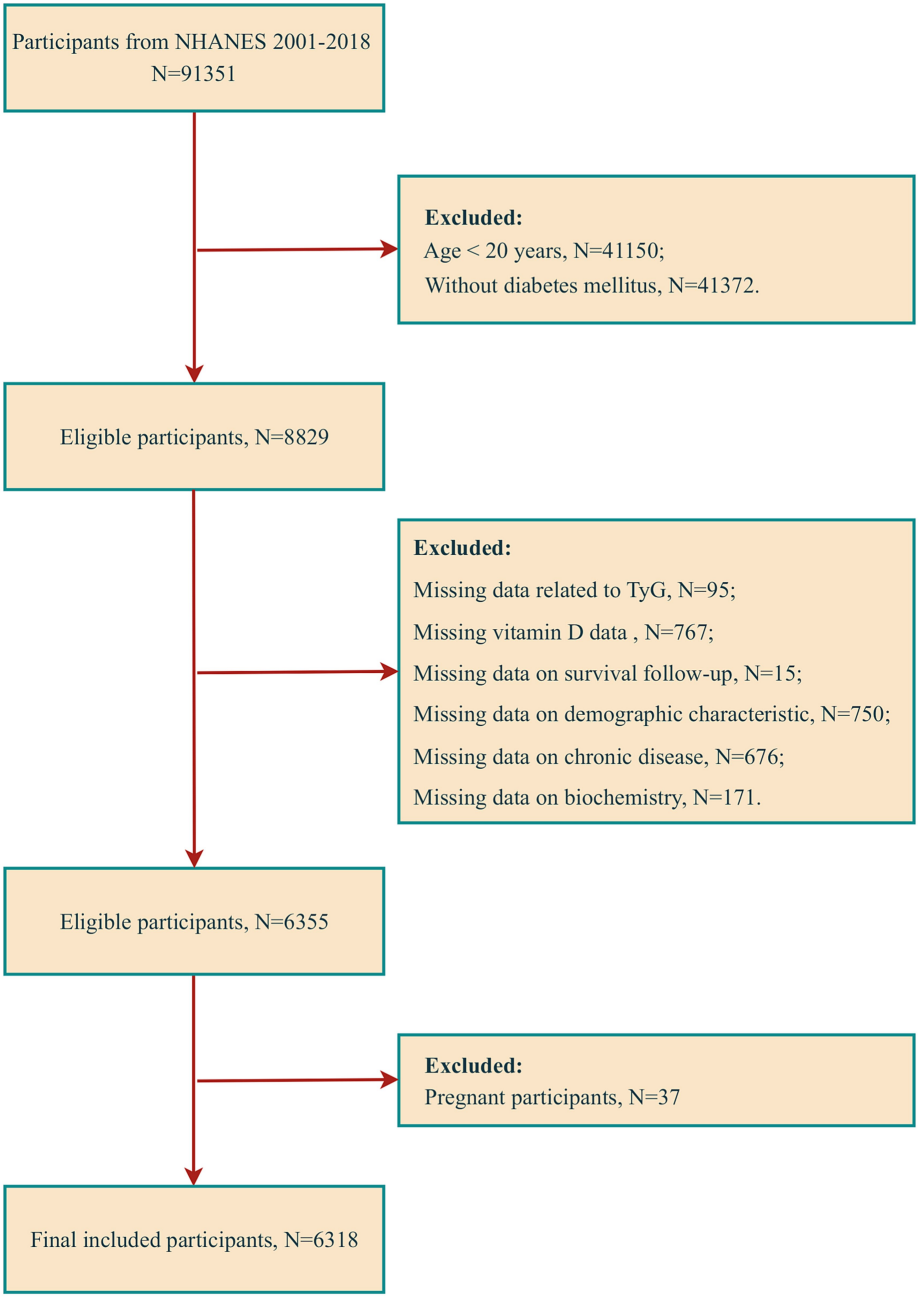
Participant screening flowchart.

The data utilized in this study were obtained from the NHANES database, spanning 2001 to 2018. This database contains the results of cross-sectional surveys conducted every 2 years by the Centers for Disease Control and Prevention (CDC). At the study’s outset, an extensive sample population was drawn from nine consecutive survey cycles, comprising 91,351 participants. DM was operationalized in this study according to the following criteria: (1) a definitive diagnosis by a healthcare professional, (2) an FPG level of 126 mg/dL or higher, (3) a glycosylated hemoglobin (HbA1c) level of 6.5% or greater, and (4) the individual’s current use of diabetic medication or insulin therapy. To ensure the accuracy and relevance of the findings, we excluded ineligible participants, including those under the age of 20, non-diabetic individuals, and those with missing data (specifically missing data on relevant indicators for calculating TyG, vitamin D, survival follow-up, demographic characteristics, chronic disease status, and some biochemical data), as well as pregnant participants. Following applying the above screening criteria, 6,318 participants were identified as eligible for inclusion in the analysis (Figure 1).

### 2.2 Ethics and STROBE statement

The research protocol of the NHANES project adhered strictly to the guidelines set forth by the Ethics Review Committee of the National Center for Health Statistics (NCHS), and all participants were required to sign an informed consent form. During data analysis, the NIH policy regulations were adhered to. Considering the anonymity and non-direct contact nature of the data, it was utilized directly in the study without requiring additional ethical review. To ensure the highest study design and reporting quality, the study was conducted according to the standards outlined in the Strengthening the Reporting of Observational Studies in Epidemiology (STROBE) statement.

### 2.3 Assessment of the TyG index

After a minimum 8.5-h fast, TG and FPG were measured using automated biochemical analyzers to ensure accurate data. FPG and TG concentrations were measured by standardized procedures. The following scientifically validated formula was employed to calculate the TyG index (3):


$$TyG=Ln\left[TG\;\left(\mathrm{mg}/\mathrm{dL}\right)\times FPG\;\left(\mathrm{mg}/\mathrm{dL}\right)/2\right]\text{.}$$

### 2.4 Vitamin D assessment

Serum 25(OH)D concentrations from NHANES 2001–2006 were measured using a Dia-Sorin radioimmunoassay kit (Stillwater, MN, United States), and serum 25(OH)D concentrations from NHANES 2007–2018 were calculated using standardized liquid chromatography–tandem mass spectrometry (LC–MS/MS). The serum 25(OH)D data from NHANES 2001–2006 have been converted by regression to equivalent 25(OH)D measurements in standardized LC–MS/MS. The conversion formulae are set forth in Supplementary material. In this study, the variable used for assessing participants’ vitamin D status was total 25OHD (sum of 25OHD2 and 25OHD3). By the Endocrine Society Clinical Practice Guidelines, the participants were categorized into two groups according to their serum 25(OH)D concentration: an insufficient group (< 75.0 nmol/L) and an sufficient group (≥75.0 nmol/L) (17).

### 2.5 Mortality assessment

The principal findings of this study pertain to all-cause mortality and cardiovascular mortality in patients with DM. All-cause mortality was defined as deaths from heart disease, malignant neoplasms, and all other causes. Disease-specific mortality was determined based on the International Statistical Classification of Diseases, 10th edition (ICD-10). Cardiovascular mortality was defined as deaths due to heart disease (ICD-10 codes 100–109, 111, 113, 120–151) and cerebrovascular disease (ICD-10 codes 160–169). The mortality data for the follow-up population were obtained from the NHANES Public Use-Related Mortality File (as of December 31, 2019). This file is correlated to the NCHS and the National Death Index (NDI) through a probabilistic matching algorithm (18). The follow-up period was calculated from the initial interview to the date of the patient’s death or December 31, 2019.

### 2.6 Assessment of covariates

In examining the correlation between TyG and vitamin D levels with all-cause and cardiovascular mortality in patients with DM, we developed multivariable-adjusted models to mitigate the influence of confounding variables on this relationship. The covariates included in this study were gender, age, race, education, marital status, family economic status, alcohol intake, smoking behavior, physical activity level, and history of several important chronic diseases, including hypertension, coronary heart disease, and stroke. For racial categorization, participants were subdivided into the following groups: Mexican American, Non-Hispanic White, Non-Hispanic Black, and Other Race. The sample was divided into three categories based on the years of education completed: less than 9th grade, 9th through 12th grade, and more than 12th grade. Marital status was dichotomized into cohabitation and solitude to ascertain the influence of family structure factors. To categorize family economic status, income was carefully divided into three intervals based on the Poverty-to-Income Ratio (PIR) criterion, as officially defined by the U.S. government. The intervals were designated as low (PIR ≤ 1.3), medium (PIR > 1.3 to ≤3.5), and high (PIR > 3.5). For this study, smoking and drinking habits were assessed using standardized methods. Smoking status was defined by the number of cigarettes smoked by the participant, with a minimum of 100 cigarettes throughout their lifetime, and current smoking status. Alcohol consumption was assessed by asking the participant whether they had consumed at least 12 alcoholic beverages of any type in the past year. Physical activity was classified into three categories: vigorous, moderate, and inactive. A comprehensive medical history was obtained for each participant, encompassing hypertension, coronary heart disease, and stroke. For hypertension, the medical history included whether the participant had ever been diagnosed with high blood pressure or was currently taking medication for high blood pressure. Similarly, for coronary heart disease and stroke, the medical history included whether the participant had ever been diagnosed with these conditions by a medical professional.

### 2.7 Statistical analysis

In the case of continuous variables, the Kolmogorov–Smirnov test was employed to verify the normality of the data. The mean ± standard deviation or median (25th and 75th percentile) were selected by the test results to characterize the variables in question. The data were presented in the form of frequencies and percentages for categorical variables.

To investigate the relationship between TyG and vitamin D levels with all-cause and cardiovascular mortality in patients with DM, multivariable Cox proportional hazard regression models were constructed to assess the impact of TyG and vitamin D levels and their quartiles on the risk of death in patients with DM, as indicated by the estimation of hazard ratios (HRs) and their 95% confidence intervals (CIs). Furthermore, additional comparisons were conducted between the sufficient and insufficient vitamin D groups. To ensure the accuracy of our assessment, we developed three multivariable adjustment models to control for potential confounding factors. Model 1 served as the baseline and did not incorporate any adjustments. Model 2 incorporated age, gender, and race. Model 3 further introduced education, marital status, family PIR, smoking status, drinking habit, physical activity level, and chronic diseases such as hypertension, coronary heart disease, and stroke.

A restricted cubic spline (RCS) model was employed to elucidate a potential nonlinear dose–response relationship between TyG levels and vitamin D status concerning mortality in patients with DM. In this model, TyG and vitamin D levels were treated as continuous variables, and the 5th, 35th, 65th, and 95th percentiles were selected as critical points for analysis based on their distributional properties. In the event of nonlinear associations, likelihood ratio tests were employed to ascertain the essential effects between TyG and vitamin D levels and mortality in patients with DM. We employed causal mediation analysis (CMA) to evaluate the mediating role of vitamin D in the relationship between TyG and mortality in individuals with diabetes. This approach enabled us to calculate both direct effects (the direct effect of TyG on mortality in individuals with DM after controlling for the mediating variable, vitamin D) and indirect effects (the impact of TyG on mortality in individuals with DM through vitamin D). Interaction-restricted cubic spline plots were constructed to illustrate potential interactions between TyG and vitamin D levels and mortality in patients with DM. Furthermore, subgroup analyses were conducted to stratify participants based on vitamin D levels and to explore the heterogeneity of the patterns of association between TyG and DM mortality risk in vitamin D insufficient and vitamin D sufficient subgroups.

All data analysis was conducted with the assistance of R 4.4.0 software (provided by the R Foundation at http://www.R-project.org) and SPSS version 23.0 (IBM Corporation, Armonk, New York, USA). Graphic presentations were generated using GraphPad Prism version 9.0 (GraphPad Software, United States).

## 3 Results

### 3.1 Baseline characteristics of patients with DM

A total of 6,318 diabetic patients were included in this study, of whom 4,775 were identified as survivors and 1,543 were identified as non-survivors. The non-survivor group exhibited a higher proportion of males (58.59%) than the survivor group (51.12%), and the mean age was higher as well (71.00 vs. 60.00 years). The racial distribution revealed a higher proportion of Non-Hispanic White individuals among non-survivors (54.05%) than among survivors (33.63%). Compared to survivors, non-survivors exhibited a lower educational level, a higher proportion of individuals living alone, a lower family income, and a lower prevalence of physical activity. The prevalence of smoking was significantly higher among non-survivors (61.50% vs. 48.08%). Additionally, the prevalence of hypertension, coronary heart disease, and stroke was higher in non-survivors. The mean body mass index (BMI), total cholesterol (TC), and vitamin D levels were lower in non-survivors. In contrast, the mean blood creatinine and uric acid levels were higher in non-survivors (Table 1).

**Table 1:** Baseline characteristics of participants with diabetes mellitus.

Variables	Total (*n* = 6,318)	Survivors (*n* = 4,775)	Non-survivors (*n* = 1,543)
Gender, *n* (%)			
Male	3,345 (52.94)	2,441 (51.12)	904 (58.59)
Female	2,973 (47.06)	2,334 (48.88)	639 (41.41)
Age (years)	62.00 (51.00, 71.00)	60.00 (49.00, 68.00)	71.00 (63.00, 80.00)
Race, *n* (%)			
Mexican American	1,222 (19.34)	997 (20.88)	225 (14.58)
Non-Hispanic White	2,440 (38.62)	1,606 (33.63)	834 (54.05)
Non-Hispanic Black	1,522 (24.09)	1,176 (24.63)	346 (22.42)
Other Race	1,134 (17.95)	996 (20.86)	138 (8.94)
Education Level, *n* (%)			
Less than 9th grade	1,094 (17.32)	736 (15.41)	358 (23.20)
9–12th grade	2,508 (39.70)	1832 (38.37)	676 (43.81)
More than 12th grade	2,716 (42.99)	2,207 (46.22)	509 (32.99)
Marital Status, *n* (%)			
Cohabitation	3,817 (60.41)	3,010 (63.04)	807 (52.30)
Solitude	2,501 (39.59)	1765 (36.96)	736 (47.70)
Family PIR, *n* (%)			
Low (≤1.3)	2,192 (34.69)	1,619 (33.91)	573 (37.14)
Medium (1.3–3.5)	2,562 (40.55)	1870 (39.16)	692 (44.85)
High (*>*3.5)	1,564 (24.75)	1,286 (26.93)	278 (18.02)
Smoke, *n* (%)			
Yes	3,245 (51.36)	2,296 (48.08)	949 (61.50)
No	3,073 (48.64)	2,479 (51.92)	594 (38.50)
Alcohol, *n* (%)			
Yes	3,803 (60.19)	2,868 (60.06)	935 (60.60)
No	2,515 (39.81)	1907 (39.94)	608 (39.40)
Physical activity, *n* (%)			
Inactive	2,513 (39.78)	1,669 (34.95)	844 (54.70)
Moderate	2,537 (40.16)	1972 (41.30)	565 (36.62)
Vigorous	1,268 (20.07)	1,134 (23.75)	134 (8.68)
Hypertension, *n* (%)			
Yes	4,014 (63.53)	2,894 (60.61)	1,120 (72.59)
No	2,304 (36.47)	1881 (39.39)	423 (27.41)
Coronary heart disease, *n* (%)			
Yes	649 (10.27)	350 (7.33)	299 (19.38)
No	5,669 (89.73)	4,425 (92.67)	1,244 (80.62)
Stroke, *n* (%)			
Yes	498 (7.88)	289 (6.05)	209 (13.55)
No	5,820 (92.12)	4,486 (93.95)	1,334 (86.45)
BMI (kg/m^2^)	31.00 (27.00, 36.12)	31.43 (27.46, 36.51)	29.66 (26.07, 34.49)
FPG (mg/dL)	132.00 (107.00, 170.00)	132.00 (107.00, 169.00)	133.00 (107.00, 174.00)
HbA1c (%)	6.70 (6.10, 7.80)	6.70 (6.00, 7.80)	6.70 (6.10, 7.70)
TC (mg/dL)	184.00 (156.00, 216.00)	184.00 (156.00, 217.00)	182.00 (154.00, 215.00)
TG (mg/dL)	156.00 (105.00, 235.00)	156.00 (105.00, 235.00)	154.00 (104.00, 233.00)
HDL-c (mg/dL)	45.00 (38.00, 56.00)	45.00 (38.00, 55.00)	45.00 (38.00, 56.00)
Creatinine (mg/dL)	0.90 (0.74, 1.10)	0.86 (0.71, 1.04)	1.01 (0.83, 1.30)
Uric acid (mg/dL)	5.60 (4.70, 6.70)	5.50 (4.60, 6.60)	5.90 (4.90, 7.20)
Vitamin D (nmol/L)	59.12 (43.31, 77.54)	59.75 (44.23, 77.90)	57.75 (41.95, 75.94)
TyG	9.24 (8.77, 9.81)	9.24 (8.77, 9.82)	9.26 (8.77, 9.80)

PIR, Poverty-to-income ratio; BMI, Body mass index; FPG, Fasting plasma-glucose; HbA1c, Hemoglobin A1c; TC, Total cholesterol; TG, Triglyceride; HDL-c, High density lipoprotein cholesterol; TyG, Triglyceride-glucose.

### 3.2 Relationship between TyG and mortality in diabetic patients

The association between the TyG index and all-cause and cardiovascular mortality was evaluated using three distinct models. In the unadjusted model (Model 1), there was no significant association between TyG and all-cause mortality (HR = 0.99, 95% CI: 0.93, 1.05). After adjustment for gender, age, and race (Model 2), the association of TyG with all-cause mortality was strengthened (HR = 1.16, 95% CI: 1.08, 1.24). After further adjustment for education level, marital status, family PIR, smoking, alcohol consumption, physical activity, hypertension, coronary heart disease, and stroke (Model 3), the association of TyG with all-cause mortality was slightly attenuated (HR = 1.11, 95% CI: 1.04, 1.19). Regarding cardiovascular mortality, a correlation was observed between TyG and cardiovascular mortality in model 2 (HR = 1.15, 95% CI: 1.02, 1.30). Further quartile-stratified analyses revealed a significantly elevated risk of all-cause mortality and cardiovascular mortality in the highest TyG quartile group in Models 2 and 3, adjusted for confounding factors (Table 2).

**Table 2:** Relationships between TyG and mortality in participants with diabetes mellitus.

Variables	Model 1	Model 2	Model 3
	HR (95% CI)	HR (95% CI)	HR (95% CI)
All-cause mortality			
TyG	0.99 (0.93 ~ 1.05)	1.16 (1.08 ~ 1.24)	1.11 (1.04 ~ 1.19)
Categories			
Quartile 1	1.00 (Reference)	1.00 (Reference)	1.00 (Reference)
Quartile 2	0.95 (0.82 ~ 1.09)	0.94 (0.81 ~ 1.08)	0.95 (0.82 ~ 1.09)
Quartile 3	1.03 (0.89 ~ 1.18)	1.04 (0.91 ~ 1.20)	0.99 (0.86 ~ 1.14)
Quartile 4	0.97 (0.85 ~ 1.12)	1.29 (1.11 ~ 1.49)	1.20 (1.04 ~ 1.39)
Cardiovascular mortality			
TyG	0.96 (0.86 ~ 1.07)	1.15 (1.02 ~ 1.30)	1.10 (0.98 ~ 1.25)
Categories			
Quartile 1	1.00 (Reference)	1.00 (Reference)	1.00 (Reference)
Quartile 2	0.93 (0.73 ~ 1.19)	0.94 (0.73 ~ 1.20)	0.96 (0.75 ~ 1.23)
Quartile 3	1.03 (0.81 ~ 1.31)	1.05 (0.83 ~ 1.34)	1.01 (0.79 ~ 1.29)
Quartile 4	1.00 (0.79 ~ 1.28)	1.39 (1.08 ~ 1.78)	1.31 (1.02 ~ 1.68)

Model 1: crude.

Model 2: adjusted for Gender, Age, Race.

Model 3: adjusted for Gender, Age, Race, Education Level, Marital Status, Family PIR, Smoke, Alcohol, Physical Activity, Hypertension, Coronary heart disease, Stroke.

TyG, Triglyceride-glucose; HR, Hazard ratio; CI, Confidence interval.

### 3.3 Relationship between vitamin D and mortality in diabetic patients

This study aimed to assess the association between vitamin D levels and all-cause and cardiovascular mortality in patients with diabetes. The unadjusted model 1 revealed a positive association between vitamin D levels and all-cause mortality (HR = 1.01, 95% CI: 1.01, 1.01). However, this association was reversed in both Model 2 and Model 3 after adjusting for multiple confounders (Model 2: HR = 0.99, 95% CI: 0.99, 0.99; Model 3: HR = 0.99, 95% CI: 0.99, 0.99). After adjusting for potential confounding variables (model 3), all-cause mortality was lower in patients with sufficient vitamin D levels than those with insufficient (HR = 0.89, 95% CI: 0.79, 0.99) (Table 3). Quartile-stratified analyses demonstrated that in both Model 2 and Model 3, adjusted for confounders, the risk of all-cause mortality was lower in the higher vitamin D quartile compared with the lowest quartile group (Supplementary Table S1). Furthermore, vitamin D levels were inversely correlated with cardiovascular mortality following adjustment for potential confounding variables (Model 2: HR = 0.99, 95% CI: 0.99, 0.99; Model 3: HR = 0.99, 95% CI: 0.99, 0.99) (Table 3). Similarly, the risk of cardiovascular mortality was lower in all quartiles with higher vitamin D levels compared with the lowest quartile group (Supplementary Table S1).

**Table 3:** Relationships between vitamin D and mortality in participants with diabetes mellitus.

Variables	Model 1	Model 2	Model 3
	HR (95% CI)	HR (95% CI)	HR (95% CI)
All-cause mortality			
Vitamin D	1.01 (1.01 ~ 1.01)	0.99 (0.99 ~ 0.99)	0.99 (0.99 ~ 0.99)
Insufficient	1.00 (Reference)	1.00 (Reference)	1.00 (Reference)
Sufficient	1.27 (1.14 ~ 1.43)	0.85 (0.75 ~ 0.95)	0.89 (0.79 ~ 0.99)
Cardiovascular mortality			
Vitamin D	1.00 (1.00 ~ 1.01)	0.99 (0.99 ~ 0.99)	0.99 (0.99 ~ 0.99)
Insufficient	1.00 (Reference)	1.00 (Reference)	1.00 (Reference)
Sufficient	1.35 (1.11 ~ 1.64)	0.86 (0.71 ~ 1.05)	0.92 (0.76 ~ 1.13)

Model 1: crude.

Model 2: adjusted for Gender, Age, Race.

Model 3: adjusted for Gender, Age, Race, Education Level, Marital Status, Family PIR, Smoke, Alcohol, Physical Activity, Hypertension, Coronary heart disease, Stroke.

HR, Hazard ratio; CI, Confidence interval.

### 3.4 RCS analysis

**Figure 2:**
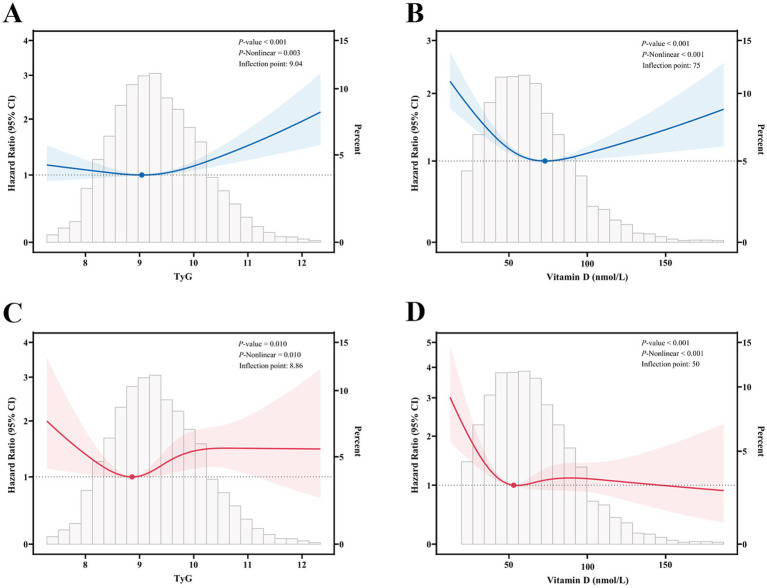
Restricted cubic spline fitting for the association between mortality and the TyG and vitamin D. **(A,B)** All-cause mortality according to the TyG and vitamin D; **(C,D)** cardiovascular mortality according to the TyG and vitamin D. Solid lines indicate HR, and shaded areas indicate 95% CI. These analyses were adjusted according to Model 3.

The results of the analysis indicated the existence of significant nonlinear associations between TyG and vitamin D levels and mortality. Figures 2A,B illustrate the correlation between TyG and vitamin D levels and all-cause mortality. Figure 2A demonstrates that the *p*-value for the overall association between TyG and all-cause mortality was less than 0.001, the nonlinear *p*-value was 0.003, and the inflection point was 9.04. This suggests that the risk of all-cause mortality initially decreased and then significantly increased with increasing TyG values. Figure 2B illustrates the overall association between vitamin D and all-cause mortality, with a *p*-value of less than 0.001, a nonlinear *p*-value of less than 0.001, and an inflection point of 75 nmol/L. This indicates a negative association between vitamin D levels and all-cause mortality at vitamin D levels below 75 nmol/L, suggesting that the risk of all-cause mortality gradually decreases with increasing vitamin D levels. Figures 2C,D illustrate the correlation between TyG and vitamin D levels and cardiovascular mortality. Figure 2C demonstrates that the p-value for the overall association between TyG and cardiovascular mortality was 0.010, the nonlinear p-value was 0.010, and the inflection point was 8.86. This suggests that the risk ratio for cardiovascular mortality increases with increasing TyG levels above 8.86. Figure 2D illustrates that the overall association between vitamin D and cardiovascular mortality was less than 0.001, the nonlinear *p*-value was less than 0.001, and the inflection point was 50 nmol/L. This suggests a significantly negative correlation between the risk ratio for cardiovascular mortality and the level of vitamin D when the level of vitamin D is below 50 nmol/L.

### 3.5 Mediating role of vitamin D in the relationship between TyG and mortality

The present study also sought to ascertain the mediating role of vitamin D in the association between TyG and mortality. The results demonstrated that vitamin D partially mediated the association between TyG and all-cause mortality, with an indirect effect of −5.82 (95% CI: −12.55, −1.71), and the proportion of mediating impact was 9.1% (95% CI: 2.3, 30.5%). However, the mediating role of vitamin D in the association between TyG and cardiovascular mortality was insignificant, with an indirect effect of −14.76 (95% CI: −38.02, −2.12). Nevertheless, the proportion of mediating effect remains uncertain (95% CI: −34.8, 84.1%) (Table 4). The analyses above were conducted after adjusting for gender, age, race, education level, marital status, family PIR, smoking, alcohol consumption, physical activity, hypertension, coronary heart disease, and stroke. These findings suggest that vitamin D may play a role in the association of TyG with all-cause mortality.

**Table 4:** Mediation of vitamin D for the association between TyG and mortality.

Mortality	Indirect effect	Direct effect	Proportion mediated, % (95% CI)
	Coefficient (95% CI)	Coefficient (95% CI)	
All-cause	−5.82 (−12.55, −1.71)	−58.24 (−152.26, −7.33)	9.1 (2.3, 30.5)
Cardiovascular	−14.76 (−38.02, −2.12)	−146.88 (−471.30, 15.57)	9.6 (−34.8, 84.1)

The mediation analyses were adjusted for Gender, Age, Race, Education Level, Marital Status, Family PIR, Smoke, Alcohol, Physical Activity, Hypertension, Coronary heart disease, and Stroke.

TyG, Triglyceride-glucose; CI, Confidence interval.

### 3.6 RCS analysis of the interaction between vitamin D and TyG

**Figure 3:**
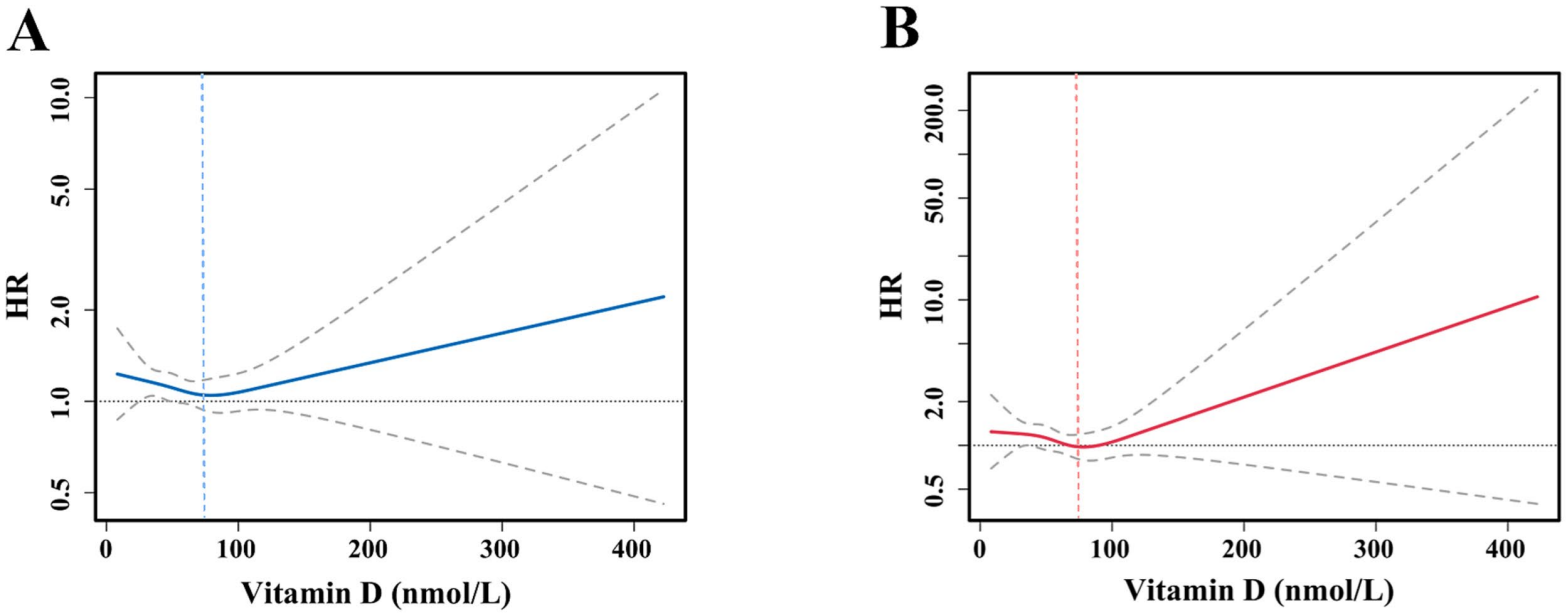
Interaction restricted cubic spline fitting of vitamin D for the association between mortality and the TyG. **(A)** All-cause mortality according to the TyG; **(B)** cardiovascular mortality according to the TyG. Solid lines indicate HR, and areas between the two dotted lines indicate 95% CI. These analyses were adjusted according to Model 3.

Figure 3A illustrates the nonlinear relationship between TyG and all-cause mortality at varying levels of vitamin D. The analyses demonstrated that alterations in vitamin D levels significantly moderated the impact of TyG on all-cause mortality. At vitamin D levels below 75 nmol/L, the HRs and 95% CIs of TyG on all-cause mortality were more significant than 1, indicating that the positive correlation between TyG and all-cause mortality was significant in this interval and gradually diminished with increasing vitamin D levels. However, when vitamin D levels were greater than or equal to 75 nmol/L, although the HR of TyG on all-cause mortality exhibited a gradual increase, the lower limit of the 95% confidence interval was markedly distant from 1, indicating that the effect of TyG on all-cause mortality was no longer statistically significant under conditions of sufficient vitamin D. Figure 3B illustrates the interaction between vitamin D and TyG levels on cardiovascular mortality. The results were analogous to those observed for all-cause mortality. When vitamin D levels were less than 75 nmol/L, the HR for cardiovascular mortality from TyG decreased progressively with increasing vitamin D levels; however, the lower limit of the 95% confidence interval was lower. The effect of TyG on cardiovascular mortality was no longer statistically significant when vitamin D levels were greater than or equal to 75 nmol/L. These findings indicate that the interaction between vitamin D and TyG may significantly prevent and treat cardiovascular disease, particularly in all-cause and cardiovascular mortality.

### 3.7 Subgroup analysis of vitamin D levels

**Figure 4:**
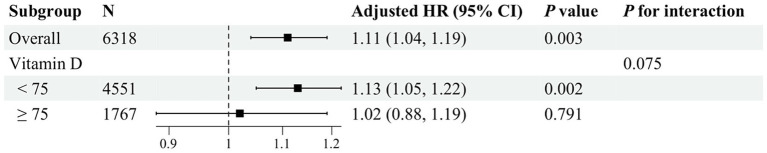
Subgroup analysis of vitamin D for the association between TyG and all-cause mortality. These analyses were adjusted according to Model 3.

**Figure 5:**
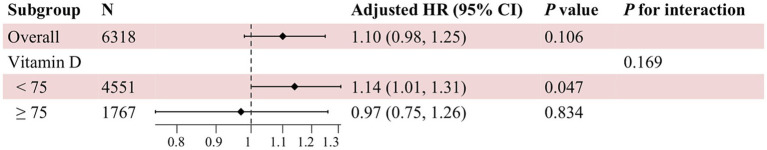
Subgroup analysis of vitamin D for the association between TyG and cardiovascular mortality. These analyses were adjusted according to Model 3.

The participants were divided into two subgroups based on their vitamin D levels, with those with levels below 75 nmol/L classified as the first subgroup and those with levels above 75 nmol/L classified as the second subgroup. The associations between TyG and all-cause and cardiovascular mortality were analyzed within each subgroup. All analyses were adjusted for potential confounding variables using a multivariable model. As illustrated in Figure 4, in the subgroup with vitamin D levels below 75 nmol/L, there was a notable elevation in the risk of all-cause mortality with each unit increase in TyG (HR = 1.13, 95% CI: 1.05, 1.22, *p* = 0.002). However, in the subgroup with vitamin D levels above 75 nmol/L, the association between TyG and all-cause mortality was attenuated. It did not reach statistical significance (HR = 1.02, 95% CI: 0.88, 1.19, *p* = 0.791). Figure 5 illustrates the correlation between TyG and cardiovascular mortality within distinct vitamin D subgroups. In the subgroup with vitamin D levels below 75 nmol/L, a significant positive correlation was observed between TyG and cardiovascular mortality (HR = 1.14, 95% CI: 1.01, 1.31, *p* = 0.047). This indicates that an increase in TyG is associated with a notable elevation in the risk of cardiovascular mortality at this vitamin D level. Nevertheless, the correlation between TyG and cardiovascular mortality was diminished and did not attain statistical significance in the subgroup with vitamin D levels above 75 nmol/L (HR = 0.97, 95% CI: 0.75, 1.26, *p* = 0.834). These findings indicate that the correlation between TyG and all-cause and cardiovascular mortality is more pronounced in individuals with insufficient vitamin D levels. Furthermore, sufficient vitamin D levels may mitigate the adverse effects of TyG on all-cause and cardiovascular mortality in diabetic patients.

## 4 Discussion

This study examined the correlation between the TyG index and all-cause mortality and cardiovascular mortality in individuals with DM across varying vitamin D levels. The analysis was conducted using data from the NHANES database. The study’s results demonstrated a statistically significant positive correlation between the TyG index and all-cause mortality and cardiovascular mortality after adjusting for potential confounding factors such as gender, age, and race. Conversely, the relationship between vitamin D level and mortality exhibited a statistically significant negative correlation, with an overall U-shaped trend. The findings indicated that the TyG index and vitamin D level were significant independent prognostic factors in diabetic patients. Further analysis of the interaction between TyG and vitamin D levels revealed that a significant positive association between TyG and all-cause mortality and cardiovascular mortality persisted in diabetic patients with insufficient vitamin D levels. In contrast, this association was significantly diminished in patients with sufficient vitamin D levels. The findings of this study offer a novel perspective on the clinical management of diabetes. Clinicians should consider both the TyG index and vitamin D levels when assessing the prognosis of diabetic patients to develop a more personalized management strategy.

Insulin resistance represents a pivotal pathophysiologic feature of diabetes and serves as a risk factor for many cardiovascular diseases and metabolic syndromes. The relationship between the TyG index, a marker for insulin resistance, and mortality in diabetic patients may be influenced by various biological mechanisms. Firstly, insulin resistance reduces the body’s sensitivity to insulin, which promotes the overproduction of glucose by the liver and increases insulin secretion, leading to hyperinsulinemia (19, 20). Chronic hyperinsulinemia may contribute to the development of atherosclerosis and increase the risk of cardiovascular disease, which in turn affects the survival of diabetic patients (20, 21). Secondly, there is an association between insulin resistance, chronic inflammation, and oxidative stress (22, 23). Increases in inflammatory factors, such as tumor necrosis factor-alpha and interleukin 6, can further exacerbate insulin resistance, promoting thrombosis and atherosclerosis. Furthermore, insulin resistance may influence the functionality and structure of microvessels, potentially leading to microvascular complications such as diabetic retinopathy and diabetic nephropathy, which could indirectly elevate the risk of mortality in patients (24, 25). Furthermore, insulin resistance frequently occurs alongside hypertriglyceridemia and low high-density lipoprotein cholesterol levels. These dyslipidemias represent significant risk factors for atherosclerosis, thereby exacerbating cardiovascular complications (26, 27). The results of the present study demonstrated a significant positive correlation between TyG and all-cause mortality and cardiovascular mortality in diabetic patients. This finding underscores the pivotal role of insulin resistance in the prognosis of diabetic patients.

It is hypothesized that vitamin D may act on mortality in diabetic patients through several mechanisms. Firstly, vitamin D may increase insulin secretion and improve insulin resistance by directly acting on pancreatic beta cells, promoting their function and proliferation (28, 29). Secondly, vitamin D has been demonstrated to inhibit the proliferation and migration of vascular smooth muscle cells, thereby reducing the formation of atherosclerosis (30, 31). Furthermore, it regulates the production of vasodilatory factors, improves vascular endothelial function, and protects the cardiovascular system from damage (32). Moreover, vitamin D exerts notable anti-inflammatory and immunomodulatory effects, inhibiting the synthesis of pro-inflammatory cytokines and mitigating the detrimental effects of inflammatory responses on the body (33, 34). Moreover, vitamin D regulates the renin-angiotensin system (RAS), which benefits blood pressure control and cardiovascular health (35–37). The present study revealed a significant negative correlation between vitamin D levels, all-cause mortality, and cardiovascular mortality in diabetic patients. These findings indicate that vitamin D levels may benefit from reducing the mortality risk in diabetic patients.

Vitamin D may influence the state of insulin resistance, and thus the risk of diabetes and its complications, through some mechanisms. First, vitamin D affects gene expression by binding to the vitamin D receptor (VDR) through its active form, 1,25-dihydroxyvitamin D. The VDR is expressed in several tissues, including muscle, fat, and liver, which play a pivotal role in insulin action and glucose metabolism. Vitamin D regulates insulin signaling in these tissues via the VDR, which may result in enhanced insulin sensitivity (38, 39). Secondly, vitamin D has been demonstrated to promote pancreatic *β*-cell proliferation and insulin synthesis and secretion, thereby contributing to normal blood glucose levels. In a state of insulin resistance, the function of pancreatic β-cells may be compromised. Vitamin D supplementation may help protect and improve these cells’ function (28, 29). Furthermore, insulin resistance is linked to a sustained, low-grade inflammatory condition. Vitamin D has anti-inflammatory and antioxidant properties that reduce inflammatory factors and oxidative stress products in the body, thereby protecting the vascular endothelium and other target organs from damage (33, 34). Furthermore, it has been demonstrated to attenuate the adverse effects of insulin resistance on mortality (40, 41). Moreover, vitamin D may impact the distribution and metabolism of adipose tissue by regulating adipocyte differentiation and function. In states of insulin resistance, the abnormal distribution and function of adipose tissue may result in metabolic disturbances. Vitamin D supplementation may prove an effective means of alleviating these conditions. Furthermore, vitamin D plays a role in regulating lipid metabolism, including triglyceride and cholesterol metabolism (41, 42). Vitamin D supplementation may facilitate improvements in blood lipid levels and a reduction in the risk of atherosclerosis, which is crucial for mitigating the risk of insulin resistance and diabetic complications (31).

The present study employed RCS analysis and subgroup analysis to ascertain whether vitamin D levels significantly modulate the effect of the TyG index on mortality in diabetic patients. The results indicated that this was indeed the case. This finding suggests vitamin D may reduce mortality risk in diabetic patients by improving insulin resistance status, thereby underscoring the synergistic role of vitamin D and insulin resistance in diabetes prognosis. The interaction between vitamin D and insulin resistance collectively influences the risk of developing diabetes and its associated complications, as well as mortality in diabetic patients. It is, therefore, of particular importance in clinical practice to develop individualized management strategies that consider the patient’s vitamin D level and insulin resistance status.

In this study, the confidence intervals observed for the mediation effect analysis for direct effects, indirect effects, and mediation proportions were notably wide. The primary potential cause for this phenomenon is the presence of errors in the measurement of the variables, which may impact the precision of the effect estimates, resulting in wider confidence intervals. The presence of unrecognized or uncontrolled confounding factors (e.g., genetic factors, dietary habits, etc.) may affect the accurate estimate of the relationship between the variables, leading to wider confidence intervals. The distribution of the variables included in the study is abnormal, which may affect the stability of the effect estimates and lead to wider confidence intervals. Additionally, the sample size included in the study may still be insufficient, which may also affect the confidence intervals. These factors may act individually or in combination to lead to the emergence of broader confidence intervals. Therefore, caution and consideration of possible limitations and uncertainties are needed when interpreting these results.

While this study offers valuable insights, its limitations may impact the interpretation and generalizability of the results. First, this study did not account for certain important confounding factors, which may have affected the accuracy of the observed results. In particular, an individual’s socioeconomic status, dietary habits, and genetic predispositions may exert an influence on the mortality rate among patients with diabetes. Socioeconomic status represents a significant confounding factor, as it is strongly associated with the complications of diabetes and may also influence an individual’s capacity to access healthcare resources. It is also possible that dietary habits, genetic factors, and other such variables may significantly impact the study outcome. However, these factors were not considered in this study. The failure to include these confounding factors may have resulted in an inaccurate estimation of the relationship between exposure and outcome, thereby limiting the reliability of the study findings. Secondly, some participants were excluded from the final analysis due to the absence of pertinent exposure variable data (e.g., vitamin D levels). This situation may have impacted the accuracy and generalizability of the study results. In particular, excluding these participants may have resulted in a biased sample selection, which may have introduced random error. If these excluded participants differed significantly from those included in the analysis regarding key characteristics such as age, gender, and lifestyle, such differences could result in a biased representation of the findings, limiting the broad applicability of the conclusions. Furthermore, missing data may introduce a systematic error, particularly if the pattern of missing data is associated with specific identifiable factors (e.g., socioeconomic status, disease severity). For instance, if participants with incomplete vitamin D data are predominantly from a lower socioeconomic status population, which may exhibit disparate health outcomes compared to the overall population, the study results may be susceptible to systematic bias. Such bias may compromise the reliability of the study findings and limit their applicability to populations from different socioeconomic backgrounds. Consequently, the potential errors associated with missing data should be fully considered when interpreting the results of this study. Future studies should prioritize the completeness and representativeness of data collection to validate further and extend the findings of this study. Furthermore, information bias represents a potential limitation of this study. In this study, information bias emerged due to measurement errors in the TyG index and vitamin D levels and inaccurate reporting of mortality data. For instance, it is conceivable that serum vitamin D measurements may not accurately reflect lifetime exposure or exposure during critical or sensitive periods. Furthermore, the mortality data collection relies on follow-up surveys, which may be subject to incomplete or inaccurate follow-up, potentially introducing additional bias. Such biases may obscure or amplify the relationship between exposure and outcome, further compromising the precision of study conclusions. To mitigate the influence of these biases, future studies should implement more rigorous sampling techniques to guarantee sample representativeness and utilize more precise measurement instruments and data collection methods to enhance data quality. Concurrently, incorporating additional confounding variables and applying advanced statistical techniques to regulate the impact of these variables is essential to attain more precise and reliable research conclusions.

## 5 Conclusion

This study examined the impact of the TyG index and vitamin D levels and their interaction on all-cause mortality and cardiovascular mortality in individuals with diabetes. The analysis was conducted using data from the NHANES database. The study’s findings indicated that TyG, as a marker of insulin resistance, was positively correlated with mortality in diabetic patients, particularly in low vitamin D levels. The beneficial effects of vitamin D on mortality in diabetic patients may be mediated by its ability to improve insulin sensitivity, exert anti-inflammatory effects, and provide cardiovascular protection, thereby attenuating the adverse impact of TyG (insulin resistance) on mortality. Moreover, the mediating effect of vitamin D levels on the relationship between TyG and mortality indicates that vitamin D may serve as a pivotal protective factor in the pathophysiological process of diabetes. These findings underscore the necessity of monitoring and adjusting TyG and vitamin D levels in diabetes management and offer novel insights for future clinical research on improving the prognosis of diabetic patients by intervening in these biomarkers. While this study is limited in scope, the results offer insight into the potential for personalized treatment and prevention strategies for diabetes. Further investigation into the interactions between these biomarkers and their potential application in diabetes management is warranted.

## Data Availability

The datasets presented in this study can be found in online repositories. The names of the repository/repositories and accession number(s) can be found in the article/Supplementary material.
