# Supplementary material for: The mediating role of vitamin D in the relationship between triglyceride glucose index and mortality in patients with diabetes mellitus: a causal mediation analysis
Source: Front Nutr. 2024 Dec 18;11:1492647. doi: 10.3389/fnut.2024.1492647 (PMC11688218; doi:10.3389/fnut.2024.1492647)
Supplement: Supplementary file 1 [file Table_1.DOCX]

**Supplementary Note 1: CDC 25(OH)D assay, bridging study, and regression equations**

The CDC LC-MS/MS method was used for measurement of 25(OH)D for NHANES 2007–2010. The processing of the NHANES 2007-2010 25(OH)D samples was delayed until the CDC developed and verified the LC-MS/MS method. In addition, sera from 1,448 participants with available 25(OH)D RIA data from NHANES 1988–1994 and 2001-2006 were reanalyzed using LC-MS/MS in order to perform a bridging (crossover) study to develop regression equations to predict a NHANES participant's LC-MS/MS-equivalent concentration from their previously measured un-harmonized (original) RIA value. A variety of regression models were evaluated. The CDC decided to provide regression equations for publicly available 25(OH)D data (NHANES 1988-1994, 2001-2002, 2003-2004, and 2005-2006) rather than using regressions based on single-year time periods that corresponded to laboratory assay dates, because that would require restricted access datasets available only in the NCHS Research Data Center.

The final regression equations were selected based on each model's predictive ability to convert RIA to LC-MS/MS-equivalents for NHANES 1988-1994, 2001-2002, 2003-2004, and 2005-2006. There were five separate regression equations (25(OH)D in nmol/L units):

NHANES 2001–2002, 2003–2004, and 2005–2006: ordinary least square regression equations were used

2001-2002: LC-MS/MS_equivalent_ = 6.43435 + 0.95212*RIA_original_

2003-2004: LC-MS/MS_equivalent_ = 1.72786 + 0.98284*RIA_original_

2005-2006: LC-MS/MS_equivalent_ = 8.36753 + 0.97012*RIA_original_

**TABLE S1** Relationships between Vitamin D and mortality in participants with diabetes mellitus.

| **Variables** | **Model 1** |  | **Model 2** |  | **Model 3** |
| --- | --- | --- | --- | --- | --- |
|  | **HR (95%CI)** |  | **HR (95%CI)** |  | **HR (95%CI)** |
| **All-cause mortality** | | | | | |
| Vitamin D Categories | | | | | |
| Quartile 1 | 1.00 (Reference)  0.86 (0.75 ~ 0.99)  0.88 (0.76 ~ 1.01)  1.21 (1.05 ~ 1.39) |  | 1.00 (Reference)  0.71 (0.62 ~ 0.82)  0.60 (0.52 ~ 0.69)  0.64 (0.55 ~ 0.74) |  | 1.00 (Reference)  0.73 (0.64 ~ 0.84)  0.65 (0.56 ~ 0.76)  0.71 (0.61 ~ 0.83) |
| Quartile 2 |  |  |  |  |  |
| Quartile 3 |  |  |  |  |  |
| Quartile 4 |  |  |  |  |  |
| **Cardiovascular mortality** | | | | | |
| Vitamin D Categories | | | | | |
| Quartile 1 | 1.00 (Reference)  0.86 (0.68 ~ 1.10)  0.90 (0.70 ~ 1.14)  1.30 (1.02 ~ 1.65) |  | 1.00 (Reference)  0.71 (0.55 ~ 0.90)  0.59 (0.46 ~ 0.76)  0.64 (0.50 ~ 0.83) |  | 1.00 (Reference)  0.72 (0.56 ~ 0.92)  0.65 (0.50 ~ 0.84)  0.74 (0.57 ~ 0.95) |
| Quartile 2 |  |  |  |  |  |
| Quartile 3 |  |  |  |  |  |
| Quartile 4 |  |  |  |  |  |

**Note:** Model 1: crude

Model 2: adjusted for Gender, Age, Race

Model 3: adjusted for Gender, Age, Race, Education Level, Marital Status, Family PIR, Smoke, Alcohol, Physical Activity, Hypertension, Coronary heart disease, Stroke

**Abbreviations:** HR, Hazard ratio; CI, Confidence interval.
